# Subsequent maternal sleep deprivation aggravates cognitive impairment by modulating hippocampal neuroinflammatory responses and synaptic function in maternal isoflurane‐exposed offspring mice

**DOI:** 10.1002/brb3.3610

**Published:** 2024-06-30

**Authors:** Meng‐Ying Zhang, Ru‐Meng Wei, Jun Jing, Shu‐Ren Huang, Gao‐Lin Qiu, Xiao‐Qiong Xia, Yue‐Ming Zhang, Yuan‐Hai Li

**Affiliations:** ^1^ Department of Anesthesiology the Affiliated Chaohu Hospital of Anhui Medical University Hefei Anhui P. R. China; ^2^ Department of Anesthesiology the First Affiliated Hospital of Anhui Medical University Hefei Anhui P. R. China; ^3^ Department of Neurology (Sleep Disorders) the Affiliated Chaohu Hospital of Anhui Medical University Hefei Anhui P. R. China; ^4^ Department of Anesthesiology Maanshan People's Hospital Maanshan Anhui P. R. China

**Keywords:** learning and memory, maternal isoflurane exposure, maternal sleep deprivation, neuroinflammation

## Abstract

**Introduction:**

Pregnant women may need to undergo non‐obstetric surgery under general anesthesia owing to medical needs, and pregnant women frequently experience sleep disturbances during late gestation. Preclinical studies demonstrated that maternal isoflurane exposure (MISO) or maternal sleep deprivation (MSD) contributed to cognitive impairments in offspring. Research studies in mice have revealed that SD can aggravate isoflurane‐induced cognitive deficits. However, it remains unclear whether MSD aggravates MISO‐induced cognitive deficits in offspring. The purpose of this research was to explore the combined effects of MSD and MISO on offspring cognitive function and the role of neuroinflammation and synaptic function in the process of MSD + MISO.

**Methods:**

Pregnant mice were exposed to 1.4% isoflurane by inhalation for 4 h on gestational day (GD) 14. Dams were then subjected to SD for 6 h (12:00–18:00 h) during GD15–21. At 3 months of age, the offspring mice were subjected to the Morris water maze test to assess cognitive function. Then the levels of inflammatory and anti‐inflammatory markers and synaptic function‐related proteins were assessed using molecular biology methods.

**Results:**

The results of this study demonstrated that MISO led to cognitive dysfunction, an effect that was aggravated by MSD. In addition, MSD exacerbated the maternal isoflurane inhalation, leading to an enhancement in the expression levels of interleukin (IL)‐1β, IL‐6, and tumor necrosis factor‐alpha and a reduction in the hippocampal levels of IL‐10, synaptophysin, post‐synaptic density‐95, growth‐associated protein‐43, and brain‐derived neurotrophic factor.

**Conclusion:**

Our findings revealed that MSD aggravated the cognitive deficits induced by MISO in male offspring mice, and these results were associated with neuroinflammation and alternations in synaptic function.

## Graphical abstract

Maternal sleep deprivation aggravated the cognitive deficits induced by maternal isoflurane exposure in male offspring mice, and these results were associated with neuroinflammation and alternations in synaptic function.

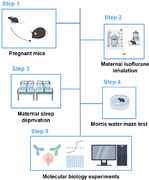

## 1 INTRODUCTION

There have been increasing numbers of pregnant women receiving non‐obstetric surgery that requires general anesthesia with surgical techniques advance recent years (Bleeser et al., 2023). Preclinical studies have also shown that isoflurane exposure during pregnancy can lead to offspring cognitive dysfunction (Fan et al., 2022). Rodent studies have documented that fetal exposure to general anesthesia induces long‐term neurobehavioral dysfunctions, such as cognitive impairment (Huang et al., 2018; Zuo, Li et al., 2020).

Nie et al. (2020) and Zhao et al. (2023) found that neuroinflammation and synaptic plasticity could contribute to cognitive deficits in the offspring mice experiencing maternal isoflurane exposure (MISO). A previous study demonstrated that upregulated levels of pro‐inflammatory cytokines (such as interleukin [IL]‐6) caused cognitive deficits in offspring mice of prenatal isoflurane exposure (Hirotsu et al., 2019). Some proteins are closely associated with synaptic plasticity and nervous system development, such as growth‐associated protein‐43 (GAP‐43), synaptophysin (SYN), and post‐synaptic density‐95 (PSD‐95) (Kobayashi et al., 1997; Thompson et al., 2008; Zou, Wei et al., 2020). GAP‐43 and SYN are localized to the pre‐synaptic membrane (Kobayashi et al., 1997; Thompson et al., 2008), whereas PSD‐95 is a major scaffolding protein found in the PSD (Qin et al., 2015). SYN and PSD‐95 affect cognitive function by influencing synaptic signal transmission and synaptic plasticity (Luo et al., 2021). In addition, GAP‐43 is one of the key factors in nerve growth and axonal regeneration. The expression of GAP‐43 can promote the extension and branching of axons, making the expression of GAP‐43 crucial for the maturation and development of cognitive function (Öhrfelt et al., 2023; Sisti et al., 2021). Studies in rats have shown that maternal exposure to isoflurane results in learning and memory impairments in offspring. Effect on MISO‐induced cognitive impairment could be partially explained by the downregulation of GAP‐43 expression (Kong et al., 2012; Nie et al., 2020). Besides, prenatal isoflurane exposure decreased the expression levels of PSD‐95 and SYN and caused cognitive deficits in offspring (Nie et al., 2020). In mice, meanwhile, isoflurane attenuated the release of brain‐derived neurotrophic factor (BDNF), a neurotrophin protein that plays a central role in neuronal survival and synaptic plasticity processes in the hippocampus (Cowansage et al., 2010), leading to learning and memory deficits (Wang, Yang et al., 2022).

Owing to the anatomical, psychological, and hormonal changes that occur in late pregnancy, mothers‐to‐be frequently suffer from sleep disorders (Facco et al., 2022). Sleep deprivation (SD) during pregnancy could induce pro‐inflammatory effects (Entringer et al., 2010) and impaired synaptic plasticity and cognitive behavior in rodents (Wei et al., 2022; Zhang, Wei, Ni et al., 2023; Zhao et al., 2015). The expression levels of pro‐inflammatory cytokines (such as IL‐1β, IL‐6, and tumor necrosis factor‐alpha [TNF‐α]) were enhanced, whereas the expression levels of anti‐inflammatory cytokines (including IL‐10) were reduced in offspring of dams exposed to maternal SD (MSD). The variations of above expression lead to diminished neurogenesis and subsequent behavioral abnormalities (Han et al., 2020; Yao et al., 2022). Furthermore, previous studies revealed that MSD markedly downregulated the hippocampal expression of synaptic plasticity–associated proteins in offspring, such as PSD‐95, SYN, and BDNF (Pardo et al., 2016; Zhang, Wei, Li et al., 2023).

MSD and MISO have been demonstrated to be important risks for cognitive impairment in offspring mice (Bleeser et al., 2021; Yu et al., 2018). A previous study showed that SD exacerbates the cognitive dysfunction in adult mice of isoflurane exposure (Zhang et al., 2020). However, the effect of MSD on prenatal isoflurane exposure has not been deeply investigated. The purpose of this study is to investigate whether MSD aggravates cognitive impairment in male offspring mice of MISO and the role of neuroinflammation and synaptic function in the process of MISO + MSD.

## 2 MATERIALS AND METHODS

### 2.1 Animals

The study protocol was approved by the Laboratory Animal Committee of Anhui Medical University (No. LLSC20190710), and the study conformed to the ARRIVE guidelines. Two‐month‐old C57BL/6J mice (Beijing Vital River Laboratory Animal Technology Co., Ltd.) were allowed to adapt to the environment for 2 weeks before the experiments. For mating, pairs of female mice were housed with a single male mouse per cage. The following day, the presence of a vaginal plug was taken as a sign of fertilization, and the day was marked as gestational day 0 (GD0). Pregnant mice were individually housed in cages in a temperature‐controlled room (22–25°C) under a 12‐h/12‐h light/dark cycle and randomly divided into four groups (eight mice per group): Control, MISO, MSD, and MISO + MSD groups. Water and food were available ad libitum.

### 2.2 Treatments

**FIGURE 1:**
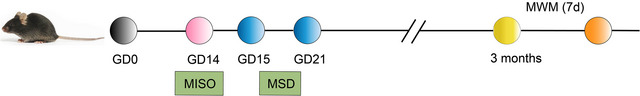
The experimental procedure of this study. GD, gestational day; MISO, maternal isoflurane exposure; MSD, maternal sleep deprivation; MWM, Morris water maze test.

On GD14, pregnant mice in MISO and MISO + MSD groups were exposed to 1.4% isoflurane (RWD Life Science Co., Ltd.) in 100% oxygen for 4 h. At the same time, pregnant mice in Control and MSD groups were exposed to 100% oxygen for 4 h (Palanisamy et al., 2011, 2017). After maternal exposure, the pregnant mice in MSD and MISO + MSD groups were undergoing SD 6 h per day for 7 days (GD15–21). After SD, the pregnant mice were put back to cage individually. The male offspring mice were separated from their dams on postnatal day 21. According to the maternal treatment factors, the male offspring mice were assigned into four groups (eight mice per group): male offspring from Control, male offspring from MISO, male offspring from MSD, and male offspring from MISO + MSD group. At 3 months of age, the behavioral and molecular biology experiments were performed to evaluate cognitive function in male offspring mice (Figure 1).

### 2.3 The MSD procedure

SD was inducing employing SD installation (BW‐NSD404, Shanghai Bio‐will Co., Ltd.). Dams in the MSD and MISO + MSD groups were individually housed in the SD installation with a running belt, and the speed of the moving belt was set at 0.5 m/min. The pregnant mice would hit the walls of the installation if they did not run on the running belts, forcing them to keep running and remain awake. All running belts operate for 6 h (12:00–18:00 h) per day for 7 days (GD15–21). At the same time, dams in Control and MISO groups were placed in the same SD with a running belt, and the belt is not moving. All mice had free access to food and water during the SD period.

### 2.4 The Morris water maze (MWM) test

Eight male offspring mice from each group were randomly selected for cognitive function evaluation using the Morris water maze (MWM) test (Wei et al., 2022). The apparatus consisted of a circular tank (radius: 75 cm, depth: 30 cm) that was divided into quadrants and filled with water (22°C). A movable platform (radius: 5 cm, height: 24 cm) was placed in one (target) quadrant. The test consisted of two phases, namely, a training phase, comprising four training sessions per day for 7 consecutive days, and a probe phase, consisting of one trial conducted after the last trial of the training phase. In the training phase, offspring mice were individually placed in the water and allowed to freely search for a hidden platform below the water surface within 60 s. Male offspring mice that did not seek the target platform within the allotted time were led to it and allowed to stay on the platform for half a minute. The ANY‐maze tracking system (Stoeling) was used to record and analyze the escape latency, swimming velocity, and distance swam. In the probe phase, the platform was removed. The animals were allowed to freely swim in the pool for 1 min. The percentage of time spent and distance swam in the target quadrant were calculated. Furthermore, the visible test was performed in the MWM test, and the results of the visible test showed that mice did not suffer from possible physical, motivational, and visual problems to find the platform.

### 2.5 Enzyme‐linked immunosorbent assays (ELISA)

After anesthesia, the mice were euthanized, and the brains were removed from the skulls. The hippocampal tissues were dissected and stored at −80°C. The frozen lysates were homogenized, centrifuged at 2000–3000 rpm for 20 min, and the supernatants were collected for measurement of the expression levels of IL‐1β, IL‐6, IL‐10, and TNF‐α. The levels of these cytokines in the hippocampus were measured using the respective enzyme‐linked immunosorbent assay (ELISA) kits (Wuhan Colorful Gene Biotechnology Co. [JYM0531Mo, JYM0012Mo, JYM0005Mo, JYM0218Mo]) according to the manufacture's protocols.

### 2.6 Real‐time fluorescence‐based quantitative PCR (qPCR)

Total RNA in hippocampal lysates of mice was extracted with TRIzol reagent (Life Technologies) and reverse transcribed into cDNA by a reverse transcription kit (Takara Bio Inc.). All quantitative PCR reactions were performed using the following conditions: one cycle of 95°C for 60 s, followed by 40 cycles of 95°C for 20 s and 60°C for 60 s. The relative gene expression levels were determined using the 2^−∆∆^ *^Ct^* method. The primer sequences are listed in Table 1.

**TABLE 1:** Primer sequences.

Gene	Forward primer (5′ → 3′)	Reverse primer (5′ → 3′)
GAPDH	GCAGTGGCAAAGTGGAGATTG	CGCTCCTGGAAGATGGTGAT
GAP‐43	GACCAAGAACATGCCTGAAC	AGGGCTCATAGGTAGGAGAG
PSD‐95	GCTCCCTGGAGAATGTGCTA	TGAGAAGCACTCCGTGAACT
SYN	GCCTACCTTCTCCACCCTTT	GCACTACCAACGTCACAGAC
BDNF	TTACTCTCCTGGGTTCCTGA	ACGTCCACTTCTGTTTCCTT

Abbreviations: BDNF, brain‐derived neurotrophic factor; GAP‐43, growth‐associated protein‐43; PSD‐95, post‐synaptic density‐95; SYN, synaptophysin.

### 2.7 Western blotting

Hippocampal tissue was lysed in RIPA buffer (Beyotime Biotechnology), and the resulting lysates were centrifuged at 12,000 × *g* for 15 min. The protein in the supernatants was separated by 10% SDS–PAGE, and then transferred to PVDF membranes, and then blocked with Western Closure Solution (5% skimmed milk powder) for 2 h. Primary rabbit anti‐GAP‐43 (dilution: 1:5000, Abcam, ab16053), rabbit anti‐BDNF (dilution: 1:1000, Boster, BA0565), rabbit anti‐PSD‐95 (dilution: 1:2000, Abcam, ab238135), rabbit anti‐SYN (dilution: 1:1000, Bioss, bs‐8845R), and mouse anti‐GAPDH (dilution: 1:2000, Zsbio, TA‐08) were subsequently used to incubate the membranes overnight at 4°C, and then membranes were incubated with HRP‐labeled goat anti‐rabbit IgG (dilution: 1:20,000, Zsbio, ZB‐2301) and goat anti‐mouse IgG (dilution: 1:20,000, Zsbio, ZB‐2305). ImageJ software (Media Cybernetics) was used to analyze images.

### 2.8 Statistical analysis

All data are expressed as means ± standard error of the mean and were tested for normality using the chi‐square test. GraphPad Prism version 8.0 was used for data analysis. Differences among groups were assessed with one‐way analysis of variance (ANOVA) or repeated measures ANOVA, followed by Tukey's post hoc test. Correlation was assessed using Pearson's correlation test. Differences were considered significant when *p*‐values were <.05.

## 3 RESULTS

### 3.1 Maternal sleep deprivation aggravated isoflurane‐induced learning and memory dysfunction in male offspring

**FIGURE 2:**
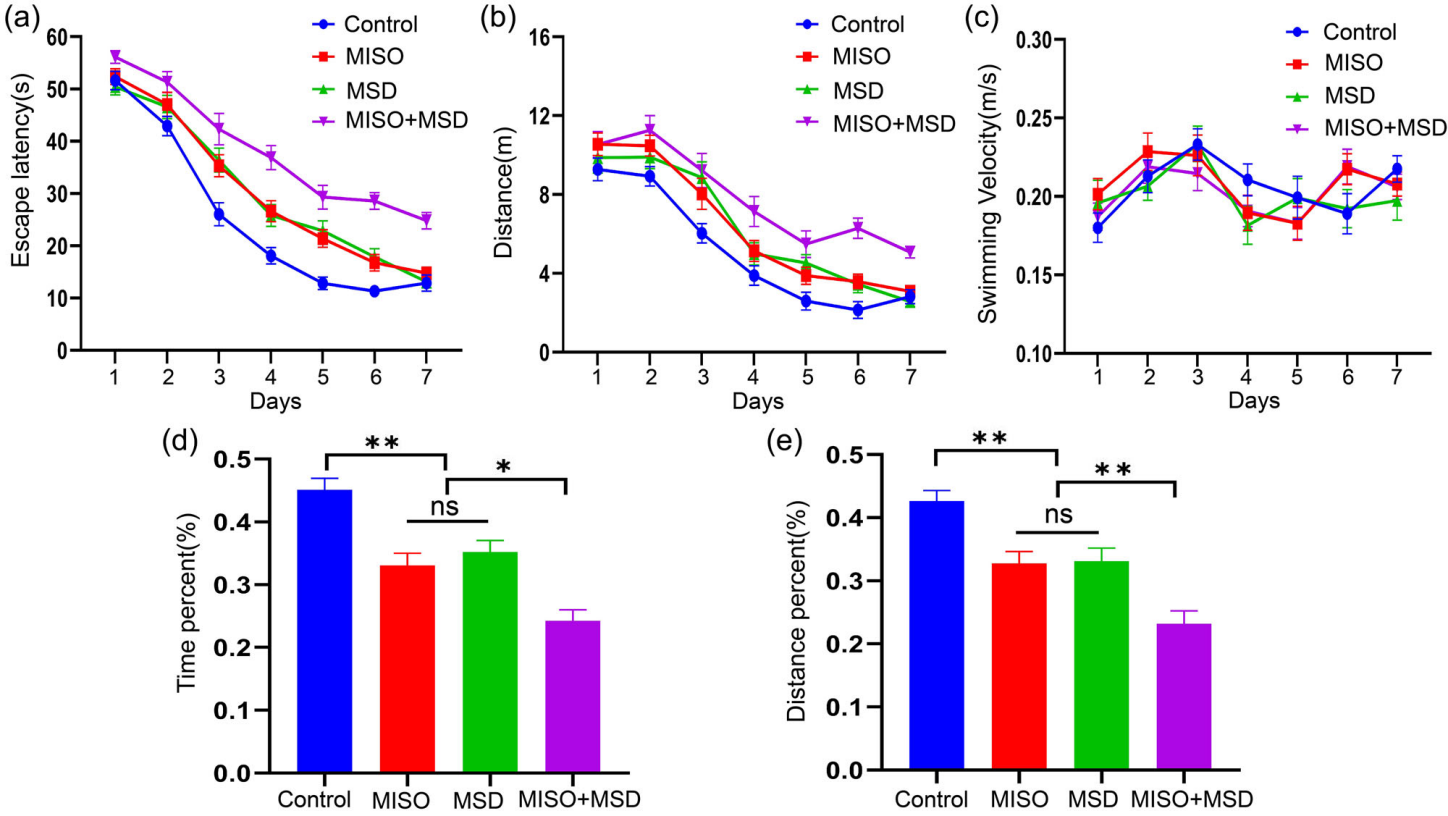
The Morris water maze test was used to evaluate learning and memory abilities: (a) escape latency; (b) distance swam; (c) swimming velocity; (d) percent time spent in the target quadrant; (e) percent distance swam in the target quadrant. *p < .05, **p < .01. MISO, maternal isoflurane exposure; MSD, maternal sleep deprivation; ns, not significant.

The MWM test was selected to determine memory and learning abilities in male offspring. In the training phase, repeated measures ANOVA showed that there were significant effects of time, treatment, and time × treatment (interaction) on latency and distance swam [latency: time: *F*_(6,168)_ = 260.80, *p* < .01; treatment: *F*_(3,28)_ = 39.42, *p* < .01; interaction: *F*_(18,168) _= 2.18, *p *> .05; distance: time: *F*_(6,168)_ = 125.0, *p* < .01; treatment: *F*_(3,28)_ = 15.44, *p* < .01; interaction: *F*_(18,168)_ = 1.25, *p* > .05]. Post hoc analysis indicated that the latency and distance were significantly prolonged in the MISO (*p* < .05) and MSD (*p *< .05) groups compared with those in the control group, whereas no significant difference was observed between the MISO and MSD groups (*p *> .05). Moreover, our data showed that the latency and distance were markedly longer in the MISO + MSD group than in the MISO group (*p *< .01) or the MSD group (*p* < .01). However, repeated measures ANOVA indicated that the swimming velocity was similar among the groups [time: *F*_(6,168)_ = 7.58, *p* < .01; treatment: *F*_(3,28)_ = .29, *p* > .05; interaction: *F*_(18,168)_ = 1.24, *p* > .05] (Figure 2a–c).

In the memory phase, there was a significant effect of treatment on the percentage of the distance and time spent in the target quadrant [percent time: *F*_(3,28)_ = 21.89, *p* < .01; percent distance: *F*_(3,28)_ = 17.10, *p* < .01]. Further analysis revealed that the percentage of the distance and time spent in the target quadrant within 1 min were significantly decreased in the MISO (*p* < .05) and MSD (*p *< .05) groups when compared with those in the control group; however, no differences were observed between the MISO and MSD groups of these parameters (*p*s > .05). Moreover, these values were further reduced in the MISO + MSD group compared with those in the MISO group (*p* < .05) or the MSD group (*p *< .05) alone (Figure 2d,e).

### 3.2 Maternal sleep deprivation aggravated isoflurane‐induced neuroinflammation in male offspring

**FIGURE 3:**
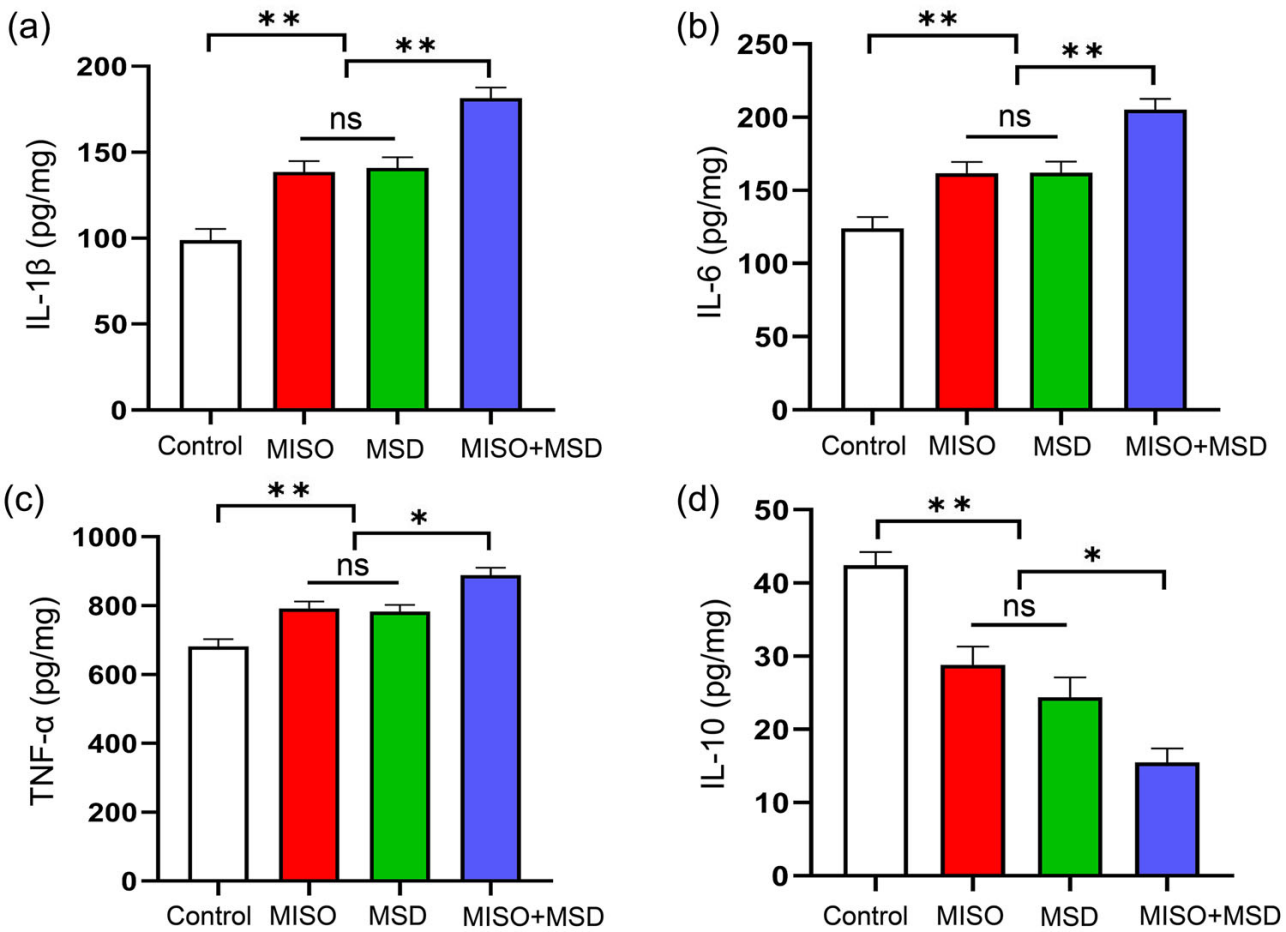
The effect of maternal sleep deprivation (MSD) on the maternal isoflurane exposure (MISO)‐induced changes in the levels of pro‐ and anti‐inflammatory cytokines in offspring mice. The hippocampal expression level of (a) interleukin (IL)‐1β, (b) IL‐6, (c) tumor necrosis factor‐alpha (TNF‐α), and (d) IL‐10. *p < .05, **p < .01. ns: not significant.

The extent of neuroinflammation in male offspring was evaluated by ELISA. The levels of IL‐1β, IL‐6, TNF‐α, and IL‐10 were significantly different among the four groups [IL‐1β: *F*_(3,28)_ = 29.29, *p* < .01; IL‐6: *F*_(3,28)_ = 19.45, *p* < .01; TNF‐α: *F*_(3,28)_ = 17.03, *p* < .01; IL‐10: *F*_(3,28)_ = 24.61, *p* < .01]. *Further* analysis suggested that MSD after isoflurane inhalation resulted in significant increases in the levels of IL‐1β, IL‐6, and TNF‐α (*p *< .01, *p *< .01, and *p *< .05, respectively) and a significant decrease in that of IL‐10 (*p* < .05) in the hippocampus compared with either the MISO group or the MSD group. However, no differences in IL‐6, TNF‐α, IL‐1β, or IL‐10 levels were detected between the MISO and MSD groups (*p* > .05) (Figure 3a–d).

### 3.3 The effect of MSD and isoflurane inhalation on GAP‐43, PSD‐95, SYN, and BDNF expression in the hippocampus

**FIGURE 4:**
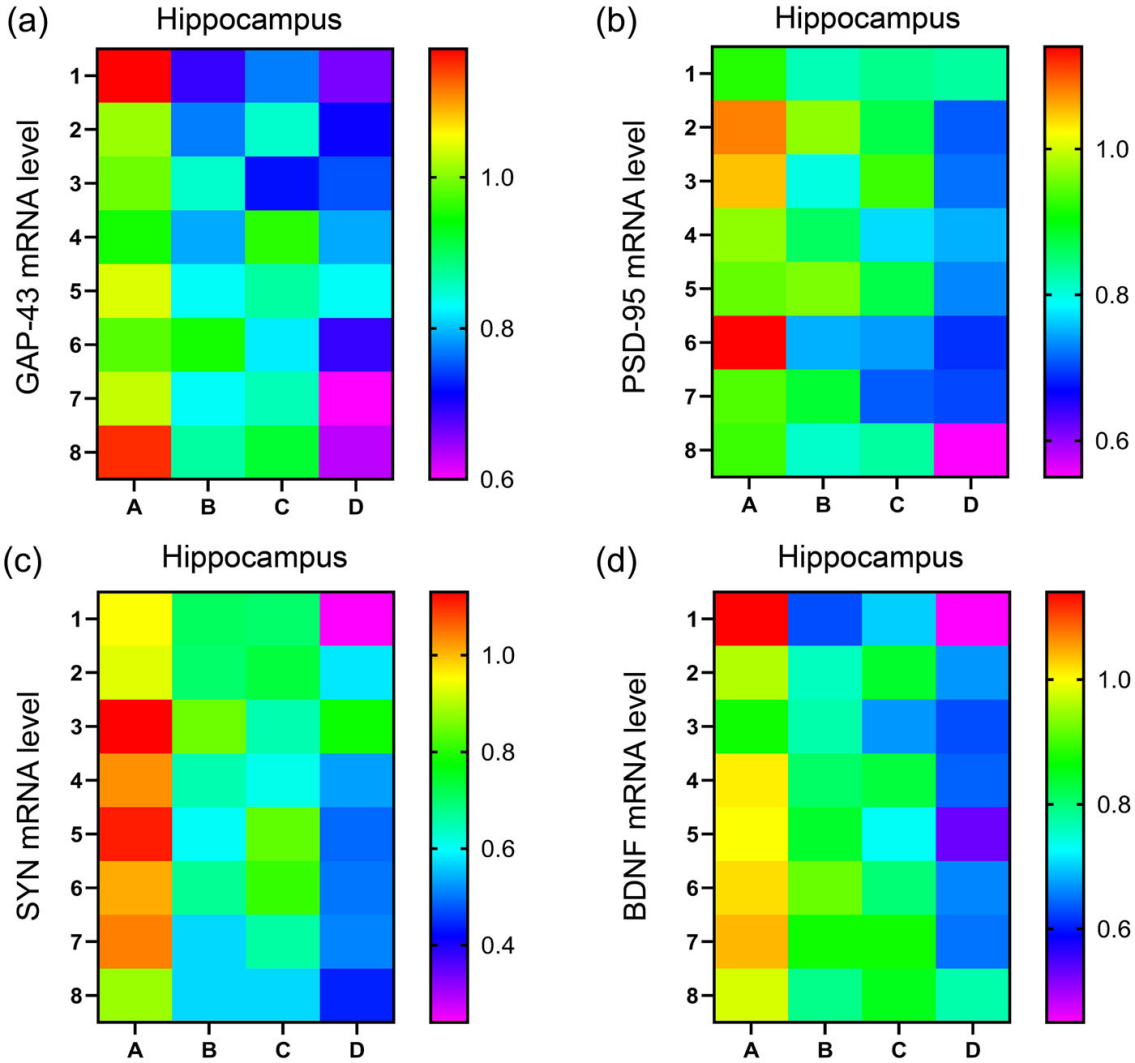
The effect of maternal sleep deprivation (MSD) on the maternal isoflurane exposure (MISO)‐induced changes in growth‐associated protein‐43 (GAP‐43), post‐synaptic density‐95 (PSD‐95), synaptophysin (SYN), and brain‐derived neurotrophic factor (BDNF) mRNA levels in the hippocampus of offspring mice. The hippocampal mRNA levels of (a) GAP‐43, (b) PSD‐95, (c) SYN, and (d) BDNF. A: control group; B: MISO group; C: MSD group; D: MISO + MSD group.

There was a significant effect of treatment on the mRNA levels of GAP‐43, PSD‐95, SYN, and BDNF [GAP‐43: *F*_(3,28)_ = 25.04, *p* < .01; PSD‐95: *F*_(3,28)_ = 18.53, *p* < .01; SYN: *F*_(3,28)_ = 29.88, *p* < .01; BDNF: *F*_(3,28)_ = 27.07, *p* < .01] in the hippocampus. Post hoc analysis suggested that the mRNA levels of GAP‐43, PSD‐95, SYN, and BDNF were significantly lower in the MISO (*p*s < .05) and MSD (*p*s < .05) groups than in the control group. However, there were no significant differences in the mRNA levels of these factors between the MISO and MSD groups (*p*s > .05). In addition, the mRNA levels of GAP‐43, PSD‐95, SYN, and BDNF in the hippocampus were further reduced in the MISO + MSD group compared with those in the MISO group (*p*s < .05) or the MSD group (*p*s < .05) (Figure 4a–d).

**FIGURE 5:**
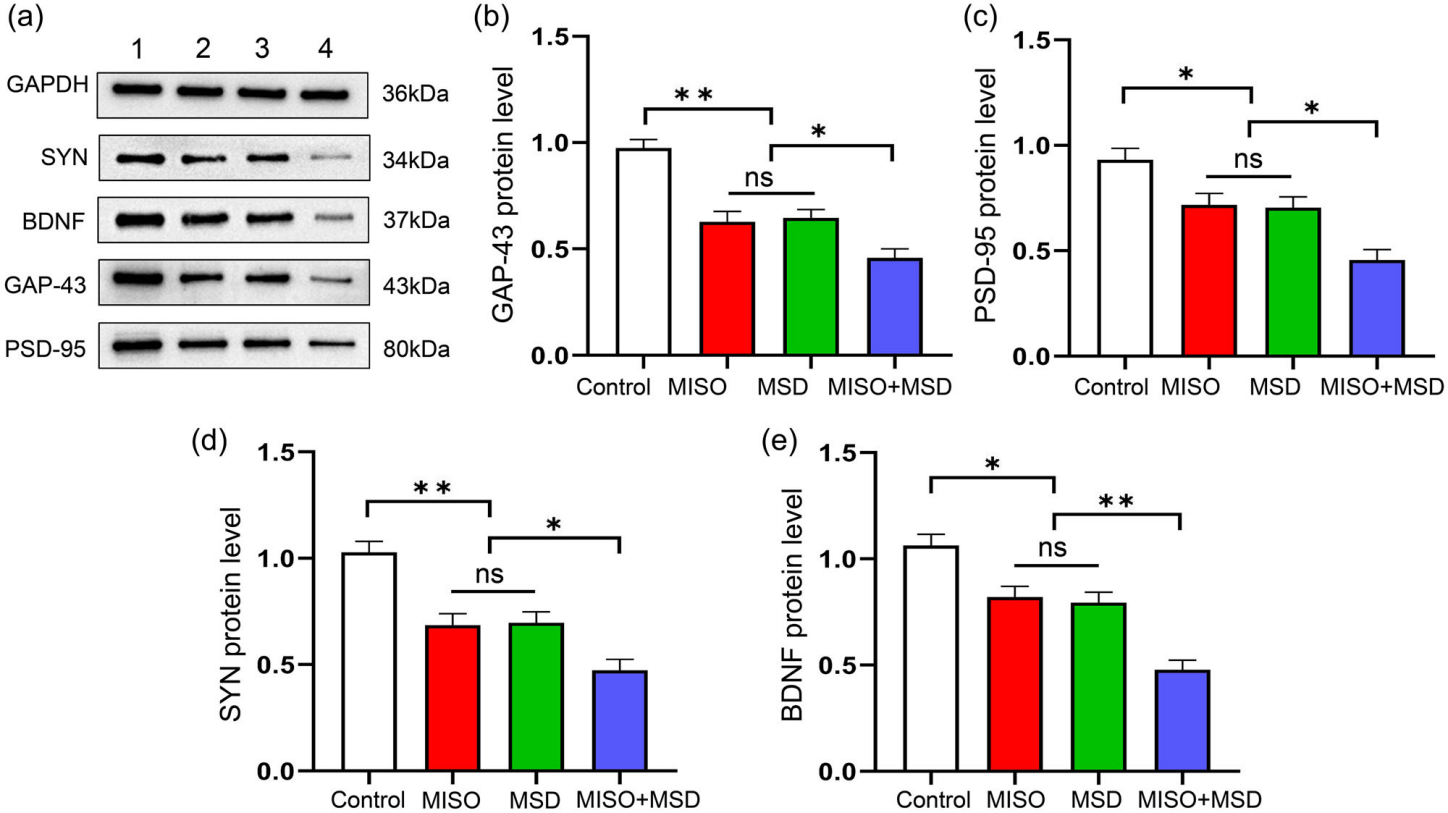
The effect of maternal sleep deprivation (MSD) on the maternal isoflurane exposure (MISO)‐induced alterations in growth‐associated protein‐43 (GAP‐43), post‐synaptic density‐95 (PSD‐95), synaptophysin (SYN), and brain‐derived neurotrophic factor (BDNF) protein levels in the hippocampus of offspring mice. (a) Western blotting: 1, control group; 2, MISO group; 3, MSD group; 4, MISO+MSD group. Hippocampal protein levels of (b) GAP‐43, (c) PSD‐95, (d) SYN, and (e) BDNF. *p < .05, **p < .01. ns, not significant.

There was a significant effect of treatment on GAP‐43, PSD‐95, SYN, and BDNF protein levels in the hippocampus [GAP‐43: *F*_(3,20)_ = 25.31, *p* < .01; PSD‐95: *F*_(3,20)_ = 13.92, *p* < .01; SYN: *F*_(3,20)_ = 19.37, *p* < .01; BDNF: *F*_(3,20)_ = 23.68, *p* < .01]. The data further showed that the protein levels of these synaptic proteins were significantly decreased in the MISO (*p*s < .05) and MSD (*p*s < .05) groups compared with those in the control group. Moreover, the expression levels of these proteins were further reduced in the MISO + MSD group compared with those in the MISO group (*p*s < .05) or the MSD group (*p*s < .05). However, the levels of the four proteins were similar between the MISO and MSD groups (*p*s > .05) (Figure 5a–e).

### 3.4 Analysis of the correlations between cognitive impairment‐like behaviors and the hippocampal expression levels of IL‐10, IL‐1β, IL‐6, and TNF‐α

Linear correlation analysis showed that the hippocampal expression levels of IL‐1β, IL‐6, and TNF‐α were positively correlated with the distance and escape latency in the learning phase of the MWM test, whereas the hippocampal levels of IL‐10 were negatively correlated with these parameters (*p*s < .05). Besides, the levels of the three pro‐inflammatory factors were negatively correlated with the percentage of the distance swam and the percentage of time spent in the target quadrant in the memory phase. However, the expression level of IL‐10 displayed a positive correlation with both parameters (*p*s < .05) (Table 2).

**TABLE 2:** Correlation between the performance in the cognition‐related tasks and hippocampal levels of inflammatory factors [*r (p)*].

Tasks	Indexes	Groups	Inflammatory factors			
			IL‐1β	IL‐6	TNF‐α	IL‐10
Morris water maze test	Escape latency	CON	−.038 (.929)	.188 (.656)	−.083 (.846)	−.056 (.895)
		MISO	.835 (.010)**	.898 (.002)**	.817 (.013)*	−.833 (.010)*
		MSD MISO + MSD	.942 (.000)** .926 (.001)**	.856 (.007)** .935 (.001)**	.852 (.007)** .881 (.004)**	−.877 (.004)** −.819 (.013)*
	Distance swam	CON	−.188 (.655)	.424 (.295)	.307 (.459)	−.108 (.799)
		MISO	.833 (.010)*	.822 (.012)*	.948 (.000)**	−.842 (.009)**
		MSD MISO + MSD	.911 (.002)** .904 (.002)**	.845 (.008)** .911 (.002)**	.852 (.007)** .882 (.004)**	−.858 (.006)** −.787 (.020)*
	Percentage of time swam	CON	−.316 (.445)	−.623 (.099)	−.491 (.216)	−.545 (.163)
		MISO	−.955 (.000)**	−.915 (.001)**	−.854 (.007)**	.757 (.030)*
		MSD MISO + MSD	−.790 (0.020)* −.933 (.001)**	−.875 (.004)** −.901 (.002)**	−.882 (.004)** −.910 (.002)**	.840 (.009)** .832 (.010)*
	Percentage of distance swam	CON	−.339 (.412)	.344 (.404)	.258 (.538)	−.067 (.875)
		MISO	−.931 (.001)**	−.952 (.000)**	−.864 (.006)**	.769 (.026)*
		MSD MISO + MSD	−.871 (.005)** −.921 (.001)**	−.911 (.002)** −.903 (.002)**	−.878 (.004)** −.862 (.006)**	.891 (.003)** .775 (.024)*

Abbreviations: CON, control; IL, interleukin; MISO, maternal isoflurane exposure; MSD, maternal sleep deprivation; TNF‐α, tumor necrosis factor‐alpha.

*P < 0.05; **P < 0.01.

### 3.5 Assessment of the correlations between cognitive impairment‐like behaviors and the hippocampal expression levels of GAP‐43, PSD‐95, SYN, and BDNF

The results showed that both the mRNA and protein levels of GAP‐43, PSD‐95, SYN, and BDNF in the hippocampus were negatively correlated with the distance swam and escape latency in the training phase (*p*s < .05). In the probe phase, meanwhile, the hippocampal contents of GAP‐43, PSD‐95, SYN, and BDNF in the four groups were positively correlated with the percentage of distance and the percentage of time spent in the target quadrant at both the mRNA and protein levels (*p*s < .05) (Tables 3 and 4).

**TABLE 3:** Correlation between the performance in the cognition‐related tasks and hippocampal mRNA levels of synaptic plasticity‐related factors [*r (p)*].

Tasks	Indexes	Groups	mRNA levels of synaptic proteins			
			BDNF	PSD‐95	SYN	GAP‐43
Morris water maze test	Escape latency	CON	.066 (.877)	−.157 (.711)	.504 (.203)	−.057 (.893)
		MISO	−.874 (.005)**	−.865 (.006)**	−.718 (.045)*	−.859 (.006)**
		MSD MISO + MSD	−.949 (.000)** −.882 (.004)**	−.741 (.036)* −.845 (.008)**	−.783 (.022)* −.906 (.002)**	−.912 (.002)** −.882 (.004)**
	Distance swam	CON	.220 (.600)	−.380 (.353)	.496 (.212)	.146 (.730)
		MISO	−.842 (.009)**	−.818 (.013)*	−.749 (.032)*	−.918 (.001)**
		MSD MISO + MSD	−.905 (.002)** −.848 (.008)**	−.708 (.050)* −.839 (.009)**	−.804 (.016)* −.884 (.004)**	−.931 (.001)** −.825 (.012)*
	Percentage of time swam	CON	.331 (.424)	−.562 (.147)	.111 (.794)	−.040 (.925)
		MISO	.815 (.014)*	.907 (.002)**	.934 (.001)**	.902 (.002)**
		MSD MISO + MSD	.788 (.020)* .900 (.002)**	.756 (.030)* .845 (.008)**	.871 (.005)** .903 (.002)**	.900 (.002)** .878 (.004)**
	Percentage of distance swam	CON	.432 (.285)	−.293 (.481)	.082 (.846)	.371 (.366)
		MISO	.748 (.033)*	.924 (.001)**	.848 (.008)**	.865 (.005)**
		MSD MISO + MSD	.887 (.003)** .838 (.009)**	.798 (.018)* .777 (.023)*	.878 (.004)** .890 (.003)**	.891 (.003)** .800 (.017)*

Abbreviations: CON, control; MISO, maternal isoflurane exposure; MSD, maternal sleep deprivation.

*P < 0.05; **P < 0.01.

**TABLE 4:** Correlation between the performance in the cognition‐related tasks and hippocampal synaptic protein levels [*r (p)*].

Tasks	Indexes	Groups	Synaptic proteins			
			BDNF	PSD‐95	SYN	GAP‐43
Morris water maze test	Escape latency	CON	.257 (.623)	.401 (.431)	.077 (.885)	.664 (.150)
		MISO	−.892 (.017)*	−.930 (.007)**	−.875 (.023)*	−.768 (.074)
		MSD MISO + MSD	−.945 (.004)** −.877 (.022)*	−.935 (.006)** −.992 (.000)**	−.984 (.000)** −.907 (.013)*	−.959 (.002)** −.860 (.028)*
	Distance	CON	.103 (.846)	.561 (.247)	−.476 (.340)	.116 (.827)
		MISO	−.925 (.008)**	−.882 (.020)*	−.992 (.000)**	−.971 (.001)**
		MSD MISO + MSD	−.863 (.027)* −.839 (.037)*	−.869 (.025)* −.967 (.002)**	−.957 (.003)** −.904 (.013)*	−.887 (.018)* −.848 (.033)*
	Percentage of time swam	CON	.633 (.177)	.218 (.678)	.215 (.683)	.501 (.311)
		MISO	.973 (.001)**	.959 (.002)**	.944 (.005)**	.854 (.031)*
		MSD MISO + MSD	.887 (.018)* .916 (.010)*	.889 (.018)* .987 (.000)**	.899 (.015)* .874 (.023)*	.901 (.014)* .921 (.009)**
	Percentage of distance swam	CON	.620 (.189)	.646 (.165)	.057 (.914)	−.155 (.769)
		MISO	.978 (.001)**	.989 (.000)**	.941 (.005)**	.889 (.018)*
		MSD MISO + MSD	.975 (.001)** .955 (.003)**	.926 (.008)** .927 (.008)**	.960 (.002)** .980 (.001)**	.958 (.003)** .911 (.011)*

Abbreviations: CON, control; MISO, maternal isoflurane exposure; MSD, maternal sleep deprivation.

*P < 0.05; **P < 0.01.

## 4 DISCUSSION

The prenatal period is critical for brain development, and, at this epoch, the embryo is especially vulnerable to environmental interference. Clinical and preclinical studies have documented that maternal anesthetics exposure can result in abnormal brain development in offspring (Huang et al., 2018; Reitman & Flood, 2011). Concurrently, pregnant women can also suffer from sleep disorders owing to alterations in physiology and anatomy that occur during pregnancy (Bacaro et al., 2020; Miller et al., 2020). However, whether sleep impairment during pregnancy exacerbates prenatal anesthetic exposure‐associated behavioral abnormalities in offspring is unknown. In the current study, we employed a new model—maternal anesthetic exposure combined with MSD—and subsequently investigated the behavioral phenotypes of offspring mice as well as the putative underlying mechanisms. The results documented that offspring mice whose dams were exposed to anesthetics or SD prenatally exhibited spatial learning and memory dysfunction. Moreover, these effects were associated with increased expression of pro‐inflammatory cytokines (IL‐1β, IL‐6, and TNF‐α) and decreased levels of anti‐inflammatory factors (IL‐10), in addition to decreases in the expression levels of markers of synaptic plasticity (BDNF, GAP‐43, PSD‐95, and SYN). Compared with either condition alone, offspring mice exposed to anesthesia in the womb and whose mothers were subjected to SD during pregnancy displayed more severe neuroinflammation and synaptic dysfunction, which was related to learning and memory deficits. Through this study, clinicians are supposed to pay attention to the sleep disorders of pregnant women during the perioperative period and actively improve the sleep problems of pregnant females to avoid the adverse effect of SD on the cognitive function in offspring.

### 4.1 Maternal sleep deprivation exacerbates cognitive dysfunction induced by prenatal isoflurane exposure

Prenatal exposure to environmental pathologic factors can lead to neuropsychiatric disorders in adulthood or old age, as proposed by the fetal origins of adult disease hypothesis (Arima & Fukuoka, 2020; Calkins & Devaskar, 2011). Emerging evidence suggests that early life stress can exert negative effects on the immune system, neuroendocrine system, and brain development (Brown, 2012; Hu et al., 2003). Clinical studies have indicated that maternal exposure to some anesthetics, including isoflurane, sevoflurane, and desflurane, in the third stage of pregnancy may impair brain development in children (Chai et al., 2020; Olutoye et al., 2018). Furthermore, the results of a preclinical study showed that offspring mice exposed to isoflurane at GD14, which is a pivotal period for brain development, showed learning and memory dysfunction in the MWM test (Kong et al., 2011). Similarly, our results revealed that maternal exposure to isoflurane led to deficits in learning and memory function in offspring mice, as reflected by the longer latency and distance in the learning phase and the lower percentage of time and distance spent in the target quadrant in the MWM test. In contrast, previous investigations showed that a single isoflurane exposure reduced the latency to find the escape hole in the Barnes maze and improved spatial learning and memory in aged mice (Butterfield et al., 2004).

Interventions applied during the perioperative period can affect the recovery of patients (James, 2023; Pestana‐Santos et al., 2021). Pregnant women must inhale anesthesia in specific situations, which can lead to sleep disorders during the perioperative period. We previously reported that MSD exacerbated the adverse effects of prenatal lipopolysaccharide exposure on cognitive function in offspring mice (Zhang, Zhang, Wei et al., 2023). In the present study, offspring mice exposed to isoflurane prenatally and whose mothers were concomitantly subjected to SD (MISO + MSD group) had longer latency and distances to locate the hidden platform in the learning phase of the MWM test, and also had a lower percentage of the total distance and time spent in the target quadrant when compared with offspring mice from the MISO group. This suggested that maternal sleep dysfunction aggravated cognitive deficits induced by prenatal isoflurane exposure. These results indicated that greater attention should be paid to pregnant women who experience perioperative sleep disorders.

### 4.2 Maternal sleep deprivation exacerbates the neuroinflammation induced by prenatal isoflurane exposure

Inflammation is a normal defense response to pathological stimulation, and an imbalanced inflammatory response is closely related to cognitive dysfunction (Ding et al., 2022; Wang, Shan et al., 2022). Accumulating evidence has indicated that stressful events during pregnancy can activate the immune system, leading to increased concentrations of pro‐inflammatory cytokines in peripheral blood (Ni et al., 2022; Solarz et al., 2023). These pro‐inflammatory factors can cross the placenta and the immature fetal blood–brain barrier and reach the fetal brain, where they induce an inflammatory response after birth (Entringer et al., 2010; Zhao et al., 2015). A previous study found that isoflurane exposure downregulated the pro‐inflammatory factors (such as IL‐18 and IL‐1β) in rats suffering from cerebral ischemia–reperfusion injury, and improved neuroinflammation and cognitive function (Zhai et al., 2023). However, one study showed that IL‐6 was significantly upregulated in the brains of offspring mice exposed to sevoflurane for 3 h on GD15.5, and this was accompanied by learning impairment, as assessed in the *Y*‐maze test (Hirotsu et al., 2019). Prenatal anesthetic exposure, a maternal stress‐inducing event, could lead to the activation of microglia in the fetus, resulting in the release of pro‐inflammatory cytokines through signaling pathways that disturb brain development in offspring mice (Zou, Wei et al., 2020). Similarly, our findings indicated that MISO significantly increased the levels of pro‐inflammatory factors (IL‐6, IL‐1β, and TNF‐α) and decreased that of the anti‐inflammatory factor IL‐10 in the hippocampus of offspring mice, which contributed to the observed spatial learning and memory impairment. MSD further increased the concentrations of pro‐inflammatory cytokines and decreased those of anti‐inflammatory cytokines following prenatal anesthetic exposure compared with those observed with prenatal anesthetic exposure alone, indicating that MSD aggravated prenatal isoflurane exposure‐induced neuroinflammation. Moreover, one study reported that chronic SD exacerbated an LPS‐induced inflammatory response by stimulating the excessive release of pro‐inflammatory cytokines in the spleen (Xu et al., 2020). Ito et al. (2020) also demonstrated that SD activated the hypothalamic–pituitary–adrenal (HPA) axis and upregulated corticosterone, which aggravated LPS‐activated inflammatory reactions (Ito et al., 2020). This suggests that the severe neuroinflammation noted in offspring may result from stronger maternal immune activation induced by SD during pregnancy. Moreover, we suspect that MSD further aggravates MISO‐activated maternal immunity via modulating the HPA reaction.

### 4.3 Maternal sleep deprivation exacerbates the synaptic dysfunction induced by prenatal isoflurane exposure

Hippocampal synaptic plasticity plays a critical role in the learning process and memory storage (Akhondzadeh, 1999; Qu et al., 2021). BDNF, the best characterized neurotrophic factor, regulates neuronal proliferation, differentiation, synaptic transmission, and synaptic plasticity through binding to TrkB receptors (Lu et al., 2014; Wang, Yang et al., 2022). In the current study, we found that prenatal isoflurane exposure decreased the BDNF expression in the hippocampus of offspring mice in adulthood, which was consistent with one study that showed that prenatal isoflurane exposure decreased the hippocampal expression of BDNF in the brains of rats 28 days after birth (Wang, Yang et al., 2022). Synaptic plasticity–associated proteins, including GAP‐43, PSD‐95, and SYN, can orchestrate synaptic maturation and may also play a role in synaptic homeostasis and plasticity. It has been reported that reduced levels of these proteins are associated with cognitive dysfunction (Farajdokht et al., 2021; Xie et al., 2023). The current study found that prenatal isoflurane exposure reduced the mRNA and protein levels of GAP‐43, PSD‐95, and SYN, suggesting that prenatal isoflurane exposure interferes with synaptic development and synaptic plasticity and subsequently leads to cognitive dysfunction in progeny. Available evidence showed that both SD and isoflurane could impair cognitive function in rodents (Han et al., 2020; Huang et al., 2018). Dendritic spines are responsible for hippocampal synaptic plasticity and vital to excitatory transmission (Rochefort & Konnerth, 2012). SD increased immature spines, and isoflurane decreased mature spines; thus, SD aggravated the cognitive impairment in isoflurane‐exposed mice (Zhang et al., 2020). In contrast, previous studies demonstrated that isoflurane enhanced the expression level of NMDA receptor 2B (NR2B) subunits and increased the long‐term potentiation (LTP) in hippocampal CA1 neurons. Due to the alterations of NR2B subunits and LTP, isoflurane exposure improved the cognitive function in mice (Rammes et al., 2009). However, the present investigation revealed that MSD further aggravated the reduction in the levels of synaptic plasticity–associated proteins and synaptic dysfunction in offspring mice.

### 4.4 Correlations between learning and memory‐associated performance and markers of inflammation and synaptic plasticity

Recent evidence supports that the expression levels of pro‐inflammatory cytokines and synaptic plasticity–associated proteins are significantly related to cognitive deficits (Zhang, Zhang, Wei et al., 2023). Upregulated concentrations of pro‐inflammatory cytokines and downregulated levels of anti‐inflammatory cytokines in the blood are associated with scores of cognitive function in patients with cognitive impairment (Huang et al., 2022). We have previously shown that the levels of pro‐inflammatory cytokines and synaptic plasticity–related proteins are strongly correlated with cognitive impairment induced by MSD (Ni et al., 2022; Wei et al., 2022). In the current study, the expression levels of pro‐ and anti‐inflammatory cytokines and synaptic plasticity–associated proteins were correlated with the parameters of the MWM test. Correlation analysis results revealed that the learning and memory deficits caused by prenatal isoflurane exposure in combination with MSD may be partly due to the neuroinflammation and synaptic dysfunction in the hippocampus; our findings also provide additional evidence that a strong relationship exists between neuroinflammation/synaptic dysfunction and the cognitive impairment induced by early life stressors.

The current study had some limitations. First, we only assessed changes in cognitive function, markers of inflammation, and synaptic plasticity–related proteins in male offspring mice, the dams of which were exposed to an anesthetic and/or SD during pregnancy; accordingly, the results may not apply to the female population. Second, changes in inflammation and protein levels were only explored in the hippocampal region, and other brain regions associated with cognitive function, such as the prefrontal cortex and the thalamus, were not examined. Finally, we did not use an intervention to suppress inflammation to observe whether the levels of behavioral indicators and synaptic plasticity–related proteins would be reversed in male offspring.

## 5 CONCLUSION

Inhalation of isoflurane at 1.4% during pregnancy negatively affects learning and memory in offspring mice, and this cognitive impairment is significantly aggravated by MSD. Furthermore, these effects are associated with neuroinflammatory responses and synaptic dysfunction. This study suggests that it is critical to pay attention to sleep patterns in pregnant women following anesthetic inhalation and, if sleep disturbances are detected, improve them as much as possible.

## AUTHOR CONTRIBUTIONS

**Meng‐Ying Zhang**: Conceptualization; investigation; methodology; validation; formal analysis; software; data curation; supervision; resources; project administration; visualization; writing—review and editing; writing—original draft. **Ru‐Meng Wei**: Conceptualization; methodology; software; formal analysis; investigation. **Jun Jing**: Supervision; visualization. **Shu‐Ren Huang**: Conceptualization; methodology; software. **Gao‐Lin Qiu**: Visualization; project administration; resources; investigation; funding acquisition. **Xiao‐Qiong Xia**: Investigation; validation; software; formal analysis. **Yue‐Ming Zhang**: Conceptualization; methodology; software; validation; formal analysis; visualization; writing—original draft. **Yuan‐Hai Li**: Writing—review and editing; writing—original draft; project administration; resources.

## CONFLICT OF INTEREST STATEMENT

The authors declare no conflicts of interest.

### PEER REVIEW

The peer review history for this article is available at https://publons.com/publon/10.1002/brb3.3610.

## Data Availability

The data that support the findings of this study are available from the corresponding author upon reasonable request.
